# NAP SACC UK: protocol for a feasibility cluster randomised controlled trial in nurseries and at home to increase physical activity and healthy eating in children aged 2–4 years

**DOI:** 10.1136/bmjopen-2015-010622

**Published:** 2016-04-06

**Authors:** R Kipping, R Jago, C Metcalfe, J White, A Papadaki, R Campbell, W Hollingworth, D Ward, S Wells, R Brockman, A Nicholson, L Moore

**Affiliations:** 1 School of Social and Community Medicine, University of Bristol, Bristol, UK; 2 Centre for Exercise, Nutrition and Health Sciences, School for Policy Studies, University of Bristol, Bristol, UK; 3 Bristol Randomised Trials Collaboration, Bristol, UK; 4 South East Wales Trials Unit, School of Medicine, Cardiff University, Cardiff, UK; 5 Department of Nutrition, Gillings School of Global Public Health, University of North Carolina at Chapel Hill, Chapel Hill, North Carolina, USA; 6 MRC/CSO Social and Public Health Sciences Unit, University of Glasgow, Glasgow, UK

**Keywords:** PUBLIC HEALTH, Physical activity, Feasibility trial, Children, Nursery

## Abstract

**Introduction:**

Systematic reviews have identified the lack of intervention studies with young children to prevent obesity. This feasibility study examines the feasibility and acceptability of adapting the Nutrition and Physical Activity Self-Assessment for Child Care (NAP SACC) intervention in the UK to inform a full-scale trial.

**Methods and analysis:**

A feasibility cluster randomised controlled trial in 12 nurseries in England, with 6 randomly assigned to the adapted NAP SACC UK intervention: nursery staff will receive training and support from an NAP SACC UK Partner to review the nursery environment (nutrition, physical activity, sedentary behaviours and oral health) and set goals for making changes. Parents will be invited to participate in a digital media-based home component to set goals for making changes in the home. As this is a feasibility study, the sample size was not based on a power calculation but will indicate the likely response rates and intracluster correlations. Measures will be assessed at baseline and 8–10 months later. We will estimate the recruitment rate of nurseries and children and adherence to the intervention and data. Nursery measurements will include the Environmental Policy Assessment and Observation score and the nursery staff's review of the nursery environment. Child measurements will include height and weight to calculate z-score body mass index (zBMI), accelerometer-determined minutes of moderate-to-vigorous physical activity per day and sedentary time, and diet using the Child and Diet Evaluation Tool. Questionnaires with nursery staff and parents will measure mediators. A process evaluation will assess fidelity of intervention delivery and views of participants.

**Ethics and dissemination:**

Ethical approval for this study was given by Wales 3 NHS Research Ethics Committee. Findings will be made available through publication in peer-reviewed journals, at conferences and to participants via the University of Bristol website. Data will be available from the University of Bristol Research Data Repository.

**Trial registration number:**

ISRCTN16287377.

**Strengths and limitations of this study**

- A feasibility trial in nurseries with children aged 2–4 years using qualitative methods to develop and adapt a US intervention for use in the UK.
- Development of a home component using digital media to involve parents.
- Mixed methods and multiple levels of assessment including environmental, self-report, objective measures, qualitative, observation and economic.
- Ability to measure the home environment with respect to nutrition, oral health, physical activity and sedentary time.
- The mediator measures need to be tested for validity and reliability.

## Introduction

There is a need to find new ways to increase physical activity and healthy eating among toddlers and preschool-aged children to reduce their risk of developing obesity and chronic diseases. In England, 22.6% of children starting primary school are overweight or obese.^1^ Internationally, the highest prevalence of childhood obesity and overweight is in the USA; however, rates in the UK and Australia remain high and the UK has one of the highest rates among European countries with Greece, Italy, Portugal and Spain having higher rates.^2^

Physical activity in children is associated with lower levels of cardiometabolic risk factors including blood lipids, blood pressure and improved psychological well-being.^3^ Physical activity patterns track moderately from childhood to adulthood indicating that physical activity is associated with short-term and longer-term health among children.^4^ In 2012, only 10% children aged 2–4 years in England were classified as meeting the current guidelines for children under 5 years,^5^ of at least 3 h of physical activity per day. Children aged 3–4 years in the UK are sedentary for an average of 10–11 h/day.^6^

Childcare settings provide opportunities to deliver interventions at the population level.^7^ Around 97% of children aged 3–4 years in England attend some form of government-funded early years education, of which 39% attend day care outside school settings.^8^ However, not all childcare settings are health-promoting environments. Assessment of physical activity in children aged 3–5 years at childcare in the USA has shown that children spend only 3% of time engaged in moderate-to-vigorous physical activity (MVPA).^9^ A study in England found that 593 children aged 4 years with valid accelerometer data met national guidelines for physical activity. However, this was mainly a light level of activity and children who attended nursery full time were more sedentary and less active in the mornings, with no differences in the rest of the day, compared with children who attended part time.^10^ The findings in this study contrast with other studies internationally which find young children not meeting national guidelines. This may reflect differences in the populations studied, as well as methods of data collection and analysis.^10^ As MVPA is closely associated with cardiorespiratory fitness and body mass index (BMI) in adolescence,^11^ it is of concern that such a small proportion of time in childcare is spent in MVPA. Further, around 80% of time at childcare is spent in sedentary activities.^9^ Childcare settings can be a strong predictor of physical activity levels and being outdoors is one of the most powerful correlates of physical activity in children.^9^ In addition, suitability of indoor play space and carer encouragement of indoor play are also predictors of MVPA.^12^ The lack of MVPA in childcare settings may be influenced by constraints of space, lack of equipment, lack of scheduled times for free play and outdoor play. A systematic review of interventions to increase physical activity in childcare settings found that regularly provided, structured physical activity programmes can increase the amount and intensity of physical activity.^13^

A diet high in fruit and vegetables and low in saturated fat has been associated with reduced risk of adult heart disease, many forms of cancer and all-cause mortality.^14^ Dietary patterns are established during childhood, yet 32% of boys and 18% of girls aged 18 months to 10 years are reported as eating no fruit during a 4-day period.^15^ Food and drink which is high in non-milk extrinsic sugars (NMES) is frequently high in calories but not in other essential nutrients and these items contribute to weight gain and tooth decay. Soft drinks contribute 14% to the intake of NMES in children aged 1–3 years and 19% to the intake of NMES in those aged 4–10 years. Saturated fat intake is also higher than the recommended 11% of total daily energy intake, at 15% for children aged 1–3 years.^15^ Preschool-aged children of low-income parents are more likely to consume table sugar and soft drinks compared with more affluent groups.^16^ In 2013, nearly a third (31%) of children aged 5 years in England, Wales and Northern Ireland had experienced obvious tooth decay in their primary teeth.^17^

Childcare centre practices and policies have been identified to have an influence on children's obesogenic dietary intake.^18^ A cross-sectional study assessing food, drink, feeding behaviour and practices in relation to national guidelines in nurseries in England found that nurseries in the most deprived areas reported serving more healthy foods (whole grains, legumes, pulses, and lentils) compared with those in less deprived areas. However, a large percentage of nurseries were not meeting national guidelines—for example, 83.7% were not serving diluted fruit juice and 71.6% were not providing oily fish every few weeks.^19^

Three systematic reviews of obesity prevention, physical activity and nutrition in young children have all identified the lack of intervention studies and the need for more research with robust study designs.^13^ ^20^ ^21^ The Cochrane review of obesity prevention in children identified a lack of effective obesity prevention interventions for children aged 0–5 years.^21^ In addition, the review recommended that studies need to better report the impact on the environment, setting and sustainability and suggested that studies testing interventions be guided by theories such as the socioecological model.^22^ Larson *et al*^20^ reviewed the regulations, practices, policies and interventions for promoting healthy eating and physical activity and for preventing obesity in children attending childcare settings. This review identified a lack of strong regulation in childcare settings in relation to health behaviours such as physical activity and diet. Yet, within childcare settings, there is ample opportunity to improve nutritional quality, time engaged in physical activity and caregivers' promotion of health behaviours. There have been a limited number of childcare interventions,^20^ and only two interventions have successfully demonstrated an effect on body weight.^23^ ^24^

The Nutrition and Physical Activity Self-Assessment for Child Care (NAP SACC) intervention was developed in the USA to fill this research and practice gap.^25^ NAP SACC aims to improve the nutrition and physical activity environment, policies and practices in childcare settings through self-assessment and targeted technical assistance. It addresses nutrition, physical activity and sedentary behaviours by giving providers a choice of where to focus change. Randomised controlled trials (RCTs) of NAP SACC in the USA have demonstrated the feasibility and acceptability of the intervention, the effectiveness of improving the environmental audit nutrition score (11% improvement from a baseline Environment and Policy Assessment and Observation (EPAO) score of 8.6),^25^ increase in nursery staff's knowledge of childhood obesity, healthy eating, personal health and working with families (all at p<0.05 level), decrease in children's z-score BMI (zBMI; p=0.02)^26^ and an increase in accelerometer-measured physical activity by 17% (p<0.05) and 46.2% increase in vigorous activity (p<0.05).^27^

**Figure 1:**
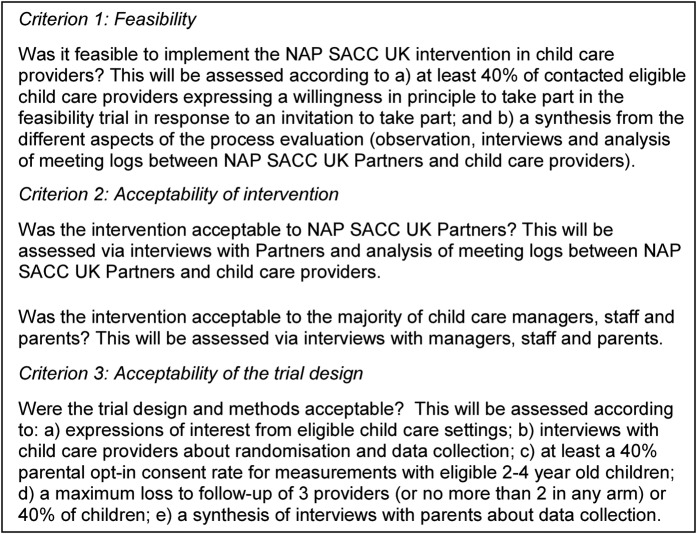
Progression criteria.

The current feasibility cluster randomised trial will use an adapted NAP SACC intervention for use in the UK, with an additional home component to involve parents, and will test the acceptability of the intervention, randomisation and the study measures. The study aims to assess whether prespecified criteria relating to the feasibility and acceptability of the intervention and trial design are met sufficiently for progression to a full-scale RCT (figure 1). Data from the study relating to the progression criteria will be assessed by the Trial Management Group (TMG) and the external Trial Steering Committee (TSC).

### Methods: participants, intervention and outcomes

The reporting of this protocol conforms to the Standard Protocol Items: Recommendations for Interventional Trials (SPIRIT) statement.^28^

#### Participants

The study will take place in 12 nurseries in two areas of England, North Somerset and Gloucestershire (with recruitment focused initially in the city of Gloucester and town of Cheltenham to ensure urban areas are included in the trial), and in the homes of children recruited to the study. North Somerset is a rural area adjacent to the City of Bristol with rural prosperity and some considerable deprivation particularly in one town, with 14.1% of children living in poverty.^29^ Gloucestershire is a large rural county to the north of Bristol, with a small city (Gloucester) and large town (Cheltenham) where the trial will be based. The health of people in Gloucestershire is generally better than the England average; however, 13.8% of children live in poverty.^30^

All children aged 3–4 years in England can access 570 h of free early education or childcare per year which is funded by the government. This is usually taken as 15 h each week for 38 weeks of the year; however, it can be accessed as 12 h/week over 48 weeks. The inclusion and exclusion criteria for nurseries, staff, children and parents/carers are as follows:

#### Inclusion criteria

- Childcare providers: Childcare settings (day nurseries, private nursery schools, maintained nursery schools, children's centres with nurseries and preschools) in North Somerset and Gloucestershire; childcare provider managers and staff recruited to the trial
- NAP SACC UK Partners: Health visitors employed in North Somerset and Gloucestershire
- Children: Children aged 2–4 years attending childcare for an average of 12 h/week across the year (or 15 h/week term time only), being provided with at least one main meal by the childcare setting
- Parents/carers: Parents/carers with children aged 2–4 years attending the providers recruited to the trial, where the child has been consented by a parent/carer

#### Exclusion criteria

- Childcare settings in North Somerset and Gloucestershire which are childminders, crèches, playgroups, primary school reception classes, where schools operate an early admission policy to admit children aged 4 years, and au pairs
- Children where the parents know the child will be leaving the childcare provider during the academic year September 2015–August 2016
- Children whose parents/carers refuse consent for measurements

#### Recruitment and consent

A range of nurseries will be recruited. Nurseries will be grouped according to location (North Somerset or Gloucestershire), Index of Multiple Deprivation (IMD) (three levels, defined separately for the two locations to have similar numbers of nurseries) and size (small or large, defined separately by a median split for the six locations by IMD combinations). IMD is a local area-based measure of deprivation in England. Nurseries in each group will be randomly chosen and invited by letter, with additional nurseries invited if a nursery declines until a total of 12 nurseries are recruited, with 6 from North Somerset and 6 from Gloucestershire. If insufficient numbers give consent from Gloucester, additional groups will be created for nurseries in Cheltenham (also within Gloucestershire). The letters will be sent from the early years leads at the two councils, with an information sheet, inviting the nursery to express interest in taking part and with an offer of meeting the research team to find out further information (see online supplementary file). Nursery managers will be asked to give consent to take part (see online supplementary file). All parents of eligible children aged 2–4 years in the recruited nurseries will be sent letters from the research team with an information sheet, inviting the parents to give opt-in consent for the child measurements (see online supplementary file). The study is aiming to recruit at least 40% of eligible children.

10.1136/bmjopen-2015-010622.supp1 Supplementary data

#### Intervention

NAP SACC is a theory-based programme that employs components of social cognitive theory (SCT) within a socioecological framework.^31^ SCT identifies the inter-relationship between the environment, people and behaviour.^32^ The socioecological framework identifies multiple, interdependent elements at policy, community, organisational, interpersonal and intrapersonal levels.^22^ Goals of the programme are to improve the nutritional quality of food served, amount and quality of physical activity, staff–child interactions and childcare settings' nutrition and physical activity policies. NAP SACC was updated in 2014 and the revised version, called ‘Go NAP SACC’, is the version which NAP SACC UK is based on without the materials for breast feeding.

**Figure 2:**
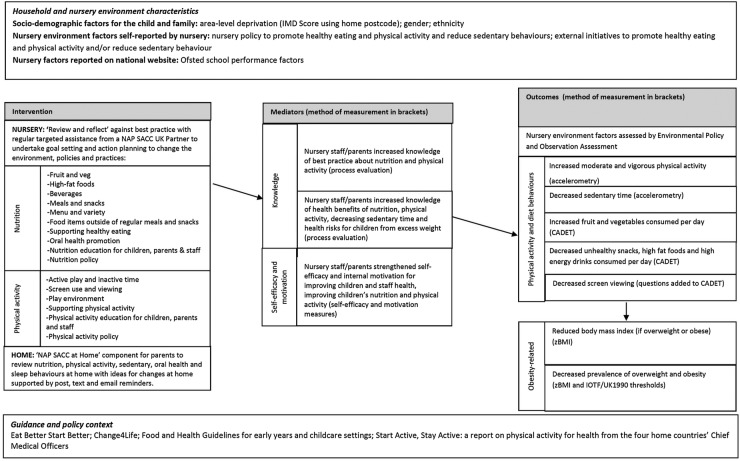
Logic model.

NAP SACC areas of focus for nutrition include fruit and vegetables; fried food and high-fat meats; beverages; menus and variety; meals and snacks; food items outside of regular meals and snacks; supporting healthy eating; nutrition education for children, parents and staff; and nutrition policy. NAP SACC areas of focus for physical activity include active play and inactive time; screen use and screen viewing; play environment; facilitating physical activity; physical activity education for children, parents and staff; and physical activity policy.^13^ The intervention used in the current trial has been adapted to reflect UK guidance on nutrition,^33^ physical activity^34^ and oral health^35^ for preschool settings and advice from dieticians. Adaptations have also been informed by focus groups or interviews with nursery managers, health visitors, public health staff, early years council staff and parents. The NAP SACC approach uses data, evidence-based action planning, choice, support, engagement and ownership, tailoring and sustained change. The logic model for the study is shown in figure 2 and steps in the intervention are outlined in box 1.

Box 1 **Steps in NAP SACC UK Intervention**

*Steps of the NAP SACC UK intervention in nurseries*

1. *Review and Reflect*: The nursery manager, together with key nursery staff, completes the Nutrition and Physical Activity Self-Assessment for Child Care UK (NAP SACC UK) Review and Reflect tool. This tool assesses the nursery on key areas in nutrition, oral health and physical activity with response options ranging from minimal to best practice.
2. *Identifying areas for improvement*: On the basis of Review and Reflect answers, facilities choose 10 areas for improvement with guidance and support from the NAP SACC UK Partner (health visitor).
3. *Workshop delivery*: A dietician and physical activity expert will deliver two half-day workshops to staff at the nursery to raise knowledge, motivation and self-efficacy to make changes. This will be followed by small group work to action plan on making improvements in the 10 areas identified through Review and Reflect process.
4. *Targeted technical assistance:* NAP SACC UK Partners (health visitors) maintain regular contact with the facility to provide support and guidance in making their improvements over 6 months.
5. *Evaluate, revise and repeat:* The NAP SACC UK Review and Reflect instrument is completed a second time to see where improvements have or have not been made. At this time, action plans are revised to include new goals and objectives and technical assistance continues.

*Steps in the NAP SACC UK at Home*

- *Sign up:* Parents are invited to sign up to take part in NAP SACC UK at Home. This involves logging onto the NAP SACC UK at Home website and registering an email address and mobile phone number for correspondence, or returning the information on paper to the NAP SACC UK office.
- *Tailoring support:* Parents are asked to complete a questionnaire about their family habits at home with respect to the areas covered by the home component to allow tailoring of support. An email or text will be sent in response suggesting areas of focus for the goals. The first 50 parents who complete the questionnaire will receive a free family swimming voucher, redeemable at local swimming pools.
- *Goal setting and action planning:* Parents will be asked to set goals for change and plan actions to meet the goals in the areas of eating, drinking, oral health, sleeping, indoor play, outdoor play, TV and screen behaviours.
- *Tailored suggestions:* Parents will receive fortnightly tips and suggestions to prompt behaviour changes in the areas where support has been requested. These will be sent via Facebook, text and emails or by post for those not online.
- *Review:* Parents will be encouraged to review their goals and actions, to consider what has worked and what could be approached differently, to set new goals and actions and consider other areas for change.

The intervention will be delivered by NAP SACC UK Partners who will all be health visitors. In England, health visitors are nurses or midwives who have received additional training. Health visitors provide a universal service to support all families while a child is aged 0–5 years, including development checks and giving information about health such as parenting, immunisation, breast feeding and weaning. Four health visitors will be recruited from the local health visiting service (based on availability rather than any pre-existing links to nurseries) and trained to work with nursery managers and staff to deliver the intervention, by supporting the nursery in the Review and Reflect process, identifying goals and actions and providing ongoing support in the changes over 6 months. The training for the NAP SACC UK Partners will be provided by local experts in nutrition, oral health and physical activity who work with childcare settings. NAP SACC UK Partner time and travel expenses will be reimbursed. Local experts in nutrition and physical activity will deliver two training sessions to nursery staff in each nursery in the intervention arm. The training will aim to raise knowledge, self-efficacy and motivation to make changes in the areas addressed by NAP SACC UK and to involve all the nursery staff in the action planning process.

In addition to the intervention in nurseries, we have developed a home component, informed by other studies of behaviour change with parents of young children^36^ use of digital media,^37^ and interviews and focus groups with parents, nursery managers and health visitors. Parents of children in the study will be invited to take part in ‘NAP SACC UK at Home’—a home component with online (via a website, text messages, emails and Facebook) support to encourage parents to make sustained changes in the home in the areas of nutrition, physical activity, sedentary behaviour, oral health and sleep with respect to their child. The steps are outlined in box 1.

Childcare providers in the control arm will continue with their usual planned activities and policies.

#### Outcomes

For the purposes of this feasibility study, the primary outcomes are the acceptability of the intervention and the trial methods. The secondary outcomes will be measured at baseline (T0), prior to the intervention and 8–10 months after the baseline (T1). Multiple visits will be made to nurseries to maximise participant retention. Assessment of the secondary outcomes will inform the choice of primary outcomes for a full-scale trial. Further, it will inform whether the outcomes require data collected from parents/children, or if the outcomes could be the environmental audit and child zBMI obtained using anonymised data linkage with the National Child Measurement Programme. The secondary outcomes to be measured will include:

1. *Environment and Policy Assessment and Observation (EPAO) instrument score*: The EPAO instrument assesses childcare nutrition and physical activity environments, policies and practices and was developed using the standards, recommendations and research literature upon which the NAP SACC intervention itself was based.^31^ It has been tested for validity and reliability in nursery settings in the USA.^38^ The EPAO consists of a 1-day observation and review of pertinent centre childcare settings' documents using 189-item questions and 16 free-text sections, with the average of all subscale scores representing total nutrition and physical activity scores. The EPAO has been adapted for use in nurseries in the UK and is administered by a researcher trained by a member of the US NAP SACC research team and blind to childcare provider allocation.
2. *Anthropometric measures of children:* zBMI and proportion of overweight and obese, as determined by the UK 1990 age and gender reference charts at 85% and 95% centiles, respectively^39^, with further sensitivity analysis using the International Obesity Task Force thresholds^40^ to facilitate international comparisons. zBMI has been demonstrated to be a good measure of change in childhood adiposity.^41^ All anthropometric measurements will be completed with children with one trained fieldworker and a member of nursery staff present. Weight will be measured without shoes in light clothing to the nearest 0.1 kg using a Seca digital scale. Height will be measured, to the nearest 0.1 cm, without shoes, using a portable Harpenden stadiometer. All measurements will be repeated and the mean measurement used. Fieldworkers will be trained to ensure correct position for height assessment.
3. *Accelerometer-measured activity* (mean minutes of sedentary, light, moderate and vigorous activity per day). We will use ActiGraph GT1M accelerometers which have been described as ‘the most widely used and extensively validated accelerometers for assessment of physical activity among children’.^42^ Accelerometers will be worn for 5 days including week and weekend days. Periods of 60 min with zero values will be interpreted as time that the monitor is not worn.^43^ A day will be considered valid if 8 h of data are recorded.^44^ Mean minutes of sedentary time (using two thresholds of 0–25 and 0–199 counts per 15 s using the criteria proposed by Evenson and Puyau^45^ ^46^ will be used to inform choice in a full trial). Mean minutes of light-intensity, moderate-intensity and vigorous-intensity physical activity will then be processed (thresholds of 200–799; and ≥800 counts per 15 s). Mean accelerometer counts per minute, which provide an indication of the overall volume of physical activity in which the children engage, will also be calculated as this approach facilitates comparison with studies that may have applied a different cut-point.
4. *Children's food and drink intake*: Dietary assessment will be performed using the Child and Diet Evaluation Tool (CADET) diary as a 24 h recall, an instrument validated for use in intervention studies with young children.^47^ ^48^ CADET will be completed by trained research staff observing food and drink consumption at nursery (to reflect diet at nursery settings), and parents will be asked to complete it for any other food and drink consumed on that day (to reflect diet at home). Parents in a sample of four nurseries will be asked to complete the CADET for 2 days at a weekend to test the feasibility of collecting weekend diet data using CADET. Parents in a sample of two nurseries will be invited to complete the CADET over the telephone to compare with sending the CADET home for parental completion.
5. *Sedentary behaviours:* In addition to the accelerometer assessments, sedentary behaviours will also be assessed by asking parents to record all screen time (TV, laptop, desktop computer, tablet, mobile phone, games console or handheld games console) and quiet play time (looking at books, playing with blocks, playing with dolls/soft toys, doing puzzles, drawing or construction) during the day the CADET tool was completed and the previous Saturday. These questions have not been validated and are based upon questions used in other studies of screen and sedentary time in preschoolers; the use of one weekday and weekend day is informed by research by Anderson *et al*.^49^ ^50^
6. *Review and Reflect tool:* Nursery staff will complete the Review and Reflect tool at the beginning and end of the intervention. This will provide an indication of the staff's assessment of any changes in the nursery environment, policy and practice relating to nutrition, physical activity, sedentary behaviour and oral health. This tool is based upon the original^13^ and revised NAP SACC self-assessment tool but has not been validated.^51^
7. *Mediators:* Parental and nursery staff knowledge (nutrition, oral health, physical activity and sedentary behaviours), self-efficacy and motivation will be assessed using tools created for this study. The reliability of the tools will be explored in a separate study to inform whether they need further refinement for use in a full-scale trial.
8. *Costs:* Nursery staff time and costs of partaking in the intervention and NAP SACC UK Partners' time and costs will be logged. Parents' direct personal costs of the child's participation in physical activity, changes in dietary patterns and health will be recorded over the previous month in a questionnaire.
9. *Quality of life:* Pediatric Quality of Life Inventory (PedsQL) for children aged 2–4 years, with 21 items which rate health-related quality of life in four domains (physical health, emotional function, social function and nursery function), will be completed by parents. Total and summary scores will be assessed. This instrument has been tested for reliability and validity in community settings.^52^

Previous research^53^ has shown that incentivising data provision is necessary for intervention and control groups; therefore, incentives will be provided for all nurseries (£200 per nursery). Children will receive a small thank-you gift (worth up to £1) for each of the two data collections. The gifts will be used in intervention and control arms. The small gift is designed to ensure that all accelerometers are returned promptly.

### Process evaluation

A process evaluation will assess the fidelity of intervention delivery calculating reach and dose and will document the views of participants about what worked well and what could be improved if we proceed to a larger trial. In addition, it will collect information about the context, facilitators and barriers to delivering the intervention.^54^ The process evaluation will include observations of the training for the health visitor and nursery staff and meetings between the NAP SACC UK Partner and each of the childcare providers. Nursery managers and NAP SACC UK Partners will be asked to complete logs of meetings including goals set, support given and progress made, and for the managers, changes made and reflections on these changes. Semistructured interviews will be conducted with all nursery managers in the providers, a sample of nursery staff, the NAP SACC UK Partners and sufficient numbers of parents until saturation is reached.

The home component will be evaluated with respect to use of the website and Facebook group, goal setting, text messages and emails using reports from the website and via semistructured interviews with parents who have different levels of engagement with the home component (none, low and high).

### Ethics and dissemination

Any protocol modifications will be submitted for ethics approval. Written informed consent will be obtained for all participants. As the effectiveness and cost-effectiveness of the intervention in the UK are unknown, we believe randomising participants to the intervention or usual care is warranted. All data will be held securely in accordance with Data Protection Regulations. Participant confidentiality will be maintained at all times. Findings will be widely disseminated in peer-reviewed journals, at conferences and to public health commissioners. Participants in the trial phase will be offered the option to receive a summary of the results once the study is complete.

### Sample size

The sample size for this feasibility study was not informed by a power calculation. The choice of 12 nurseries will provide some information on variability within and between nurseries at baseline and follow-up. This sample will not provide a usefully precise estimate of the intervention effect. However, the sample will indicate the likely response rates and intracluster correlations (ICCs) in anticipation of a larger trial. We will also use information on effect sizes and ICCs from other adequately powered diet, physical activity and obesity prevention trials to inform any calculation for a future phase III trial.

### Randomisation

Randomisation of nurseries will occur after all nurseries have completed baseline data collection. The nurseries will be the unit of allocation to two arms: NAP SACC UK or no intervention (usual practice). Allocation will be conducted by an independent statistician at the Bristol Randomised Trials Collaboration (BRTC), blind to the identity of the nurseries. Stratified randomisation will be used to ensure that the numbers of participants receiving each intervention are closely balanced within each stratum. Stratification will be based first on high/low England IMD for the local super output area where the nursery is located; the 12 selected childcare providers will be ranked by their IMD scores separately for North Somerset and Gloucestershire; the highest 3 and lowest 3 in North Somerset and highest 3 and lowest 3 in Gloucestershire will be assigned to the strata. Stratification will be based second on location (North Somerset or Gloucestershire). Childcare providers in the control arm will continue with their usual planned activities and policies. The randomisation procedure blinds all staff and casual fieldworkers to the allocation of nurseries at the baseline data collection. The trial statistician (CM) will have no contact with the nurseries, participants or the study fieldworkers.

### Data management

Data will be entered and transcribed by the research staff using a secure data management system at the University of Bristol. Completed questionnaires will be transported to the University of Bristol by the study manager or the recruited fieldworkers. Data from questionnaires will be stored in anonymised form, using participant identification numbers. Participant identification numbers and corresponding participant names will be held in separate files. Both files will be stored in secure password-protected folders. Individuals' names will be replaced with pseudonyms in interview/focus group transcripts. A list of participant names, pseudonyms and their unique identification number will be held securely in a separate location. Digital recordings of interviews/focus groups will be stored securely and will be held separately from transcripts and information on participant identities.

### Analysis

The statistical analyses for this feasibility study will be primarily descriptive, providing realistic estimates of eligibility, recruitment, intervention delivery and retention rates in the study population, with 95% CIs calculated to incorporate between-provider variation where appropriate. The CONSORT flow diagram for clinical trial reporting will be completed. Summary statistics will also be presented for the outcome measures using means and SDs by allocation arm and key demographic variables as these will also inform the sample size and recruitment plan for the main trial. Differences will be explored for each of these measures by study location (North Somerset/Gloucestershire) and deprivation (high vs low). Comparisons will be made between those who complete the study and those who drop out to investigate if this is a potential source of bias. Missing data will not be imputed for the purposes of the feasibility study. However, the extent of missing data will be examined and described to inform the full trial. Stata statistical software will be used for all analyses.

We do not plan an economic evaluation alongside this feasibility trial. Our aim is to pilot measures of resource use and estimate more precisely the cost of the intervention to inform a full-scale trial. Costs and outcomes will be presented in a cost-consequence table. We will delineate the resource use (eg, h), unit costs (eg, cost/h) and calculate mean, provider and parental costs in the intervention and control groups. We will estimate incremental costs and 95% CIs for descriptive purposes.

For the qualitative analysis, all interview recordings will be transcribed verbatim and anonymised. As the data are exploratory, we will adopt a thematic analytical approach. Meaningful content will be coded and codes grouped to form themes that describe the content of codes. Quotations which best represent the nature of each theme will then be extracted.

## Discussion

This paper describes the protocol for the NAP SACC UK feasibility trial, which is attempting to improve the nursery environment and health of children aged 2–4 years with respect to nutrition, oral health, physical activity and sedentary time. Many young children in the UK do not achieve national standards for nutrition, oral health or physical activity and, upon entry to primary school, overweight and obesity are prevalent. Childcare settings are increasingly important in the UK with 15 h/week provided free for children aged 3–4 years and the intention is to increase this to 30 h/week for children in England by 2017.^55^ Given the lack of effective interventions to increase physical activity and healthy eating in young children, and the small number of trials which have been conducted in childcare settings in the UK, this study will provide important information to inform research and practice. The goal of this feasibility trial is to assess the potential of this intervention, developed and used successfully in the USA, to be adapted for use in the UK, expanded to involve parents and to provide all the information necessary to design a cluster RCT in UK childcare settings.

### Trial status

The current study status (26 January 2016): we have obtained ethical approval for the study, funding for the study and have recruited all project staff. Nursery and child recruitment began in August 2015 and baseline data collection started in September 2015 and will be completed by mid-February 2016. NAP SACC UK Partner training took place in December 2015 and January 2016 and the intervention will start in February 2016. At the inaugural meeting of the TSC, it was agreed that a data monitoring committee was not necessary as there were no safety concerns associated with implementing NAPSACC UK and no interim analyses were planned.

### Trial governance

The principal investigator (RK) will have overall responsibility for the conduct of the study.

Day-to-day management will be coordinated by the trial manager (SW/AN) who will be closely monitored and supported by the principal investigator. A TMG is chaired monthly by the principal investigator and includes the coinvestigators and the trial manager. In addition, the principal investigator will meet with the trial manager every 2 weeks to address day-to-day issues. We will form a Local Advisory Group (LAG) of representatives from our collaborators including early years advisors in the councils, health visitors, childcare managers, childcare staff and parents. The LAG will advise on the delivery of the intervention and provide guidance on any provider-related, parent-related or child-related issues that might arise during the course of the intervention. The LAG will meet twice during the intervention year and immediately before the follow-up assessment. An independent TSC has been established. The TSC comprises professor Russell Viner (chair, University College London and London NHS Foundation Trust), professor Sian Robinson (University of Southampton), Dr Brad Metcalfe (University of Exeter), Claire Wilson (health visitor), Trudy May (nursery manager), Shelia Ogilvie (health visitor), Justine Britton (nursery manager) and Ruth Kipping (PI).

### Safety monitoring and reporting

Nursery managers and those delivering the intervention will be asked to contact the study team within 5 working days if any untoward incident or adverse event (AE) occurs to a member of staff or child, as a direct result of taking part in NAP SACC UK, or due to changes that have occurred in the nursery environment due to participation in NAP SACC UK. In these cases, study-specific AE/incident report forms will be used to record information on the event. All AE/incident report forms will be discussed with the principal investigator to assess seriousness and to confirm causality. All AEs deemed to be ‘serious’ (SAE) will be reported to the sponsor within 24 h. Where the SAE is suspected to be related to the intervention and unexpected (NB: there are no expected events for this intervention), that is, a suspected unrelated serious adverse reaction (SUSAR), the chair of the TSC and the REC will be notified within 15 days of the study team receiving the initial report.
