# Supplementary material for: NAP SACC UK: protocol for a feasibility cluster randomised controlled trial in nurseries and at home to increase physical activity and healthy eating in children aged 2–4 years
Source: BMJ Open. 2016 Apr 6;6(4):e010622. doi: 10.1136/bmjopen-2015-010622 (PMC4823443; doi:10.1136/bmjopen-2015-010622)
Supplement: Supplementary data [file bmjopen-2015-010622supp.pdf]

Supplementary File: NAP SACC UK Research Study participant information sheets and consent forms

‘NAP SACC UK Research Study’
The Nutrition and Physical Activity Self Assessment for Child Care
Name of researcher: Dr Ruth Kipping

We are doing some exciting research with nurseries and other child care providers to help them provide young children with healthy food and drink and offer opportunity to do enough exercise. As part of this research we are inviting staff in child care providers to help the study by taking part in a telephone interview. Would you be willing to take part?

INFORMATION FOR CONSENT FOR TELEPHONE INTERVIEW

Purpose
It is important for children to do some exercise and eat fruit and vegetables every day for healthy growth and development. Lots of children in England and Wales do not do enough exercise or eat enough fruit and vegetables. One in five children who start primary school are overweight or obese.

This study will aim to test whether we can adapt and use a 6 month programme from the United States called “The Nutrition and Physical Activity Self Assessment for Child Care” (NAP SACC). We will make changes to use it in the UK and test whether we can work with nurseries and other child care providers to make them healthier places for children.

We are very keen to gain child care staff’s opinions and find out what you think. We will be asking about changes which could be made so children have more opportunities to have healthy levels of exercise and a healthy diet. As part of this research, you are being invited to participate in a telephone interview with a researcher. The telephone interview will take no more than 1 hour at a time which is convenient to you. The information we gather will help us understand the thoughts, opinions and attitudes of child care staff working with preschool children, such as yourself. The information we collect will be used to help us make changes to ‘NAP SACC’ before we trial it in nurseries and other child care providers.

Participation

Your participation in the interview is voluntary. You can choose not to take part, or you may withdraw at any time.

Risks

There are no risks associated with taking part in the interview.

Benefits

The information will help us decide what changes we need to make to the programme and the research study.

Confidentiality

Everything you say during the interview will remain confidential. No information will be given to your nursery or other child care providers. No names or identifying information will be used in any results, publication or presentations. The interview session will be audio-taped. Direct quotes (things you have said) from the conversation may be used in reports, but no identifying information will be included (i.e. we will **not** include your name, or the name of your nursery or other child care provider).

Questions

If you have any further questions about the study, please contact the Principal Investigator, Dr Ruth Kipping, or one of the members of the NAP SACC UK study team on 0117 9287308 or email napsacc-uk@bristol.ac.uk. They will be happy to assist with any queries.

What to do next

If you are happy to take part in an interview please complete the reply form on the next page, ticking all relevant boxes and filling in the information requested. Return the form in the prepaid envelope enclosed. One of the research team will then contact you with the time and venue.

If you do not wish to take part in the interview you do not need to do anything, however you may receive a reminder letter in a few weeks. If you do not wish to take part please ignore this letter.

‘NAP SACC’ study

CHILD CARE STAFF INTERVIEW

REPLY AND CONSENT FORM

Name of lead researcher: Dr Ruth Kipping

Please complete all details and return in prepaid envelope:

Please put your initials in box for each question

I do wish to take part in the interview and I am happy for the NAP SACC UK team to contact me about a time for the interview	Yes	☐
The purpose of the interview has been explained to me, and I have been given the chance to discuss any questions or concerns with the researcher.	Yes	☐
I understand that the interview will be audio-taped but that my responses will remain confidential and I will not be able to be identified in any results.	Yes	☐
I agree to take part in the interview and understand that I may stop the interview at any time without giving a reason.	Yes	☐

Name of member of staff...

Job title ....

Name of nursery/child care provider ....

Address...

....

Telephone number..

Preferred time for interview: Morning ☐ Afternoon ☐ Evening ☐

Signature .... Date ………………………………

Please return form in prepaid envelope provided

NAP SACC UK, University of Bristol, Canynge Hall, 39 Whatley Rd, Bristol BS8 2PS

To be completed by researcher

Researcher name ....

Signature .... Date …………………………......

‘NAP SACC UK Research Study’
The Nutrition and Physical Activity Self Assessment for Child Care

Name of researcher: Dr Ruth Kipping

We are doing some exciting research with nurseries and other child care providers to help them provide young children with healthy food and drink and offer opportunity to do enough exercise. As part of this research we are inviting Early Years’ and Public Health staff to help the study by taking part in a focus group. Would you be willing to take part?

INFORMATION FOR CONSENT FOR FOCUS GROUP

Purpose

It is important for children to do some exercise and eat fruit and vegetables every day for healthy growth and development. Lots of children in England and Wales do not do enough exercise or eat enough fruit and vegetables. One in five children who start primary school are overweight or obese.

This study will aim to test whether we can adapt and use a 6 month programme from the United States called “The Nutrition and Physical Activity Self Assessment for Child Care” (NAP SACC). We will make changes to use it in the UK and test whether we can work with nurseries and other child care providers to make them healthier places for children.

We are very keen to talk to Early Years’ and Public Health staff to gain your opinions and find out what you think. We will be asking about changes which could be made so children have more opportunities to have healthy levels of exercise and a healthy diet. As part of this research, you are being invited to participate in a focus group, like a discussion group, with other staff and a researcher. The focus group will take no more than 1.5 hours and will take place in Clevedon for staff working in North Somerset, or Cardiff for staff working in Wales. The information we gather will help us understand the thoughts, opinions and attitudes of child care staff working with preschool children, such as yourself. The information we collect will be used to help us make changes to ‘NAP SACC’ before we trial it in nurseries and other child care providers.

Participation

Your participation in the focus group is voluntary. You can choose not to take part, or you may withdraw at any time. We will pay for your travel expenses to attend the focus group.

Risks

There are no risks associated with taking part in the focus group.

Benefits

The information will help us decide what changes we need to make to the programme and the research study.

Confidentiality

Everything you say during the focus group will remain confidential. No names or identifying information will be used in any results, publication or presentations. The interview session will be audio-taped. Direct quotes (things you have said) from the conversation may be used in reports, but no identifying information will be included (i.e. we will **not** include your name, names of nurseries or other child care providers).

Questions

If you have any further questions about the study, please contact the Principal Investigator, Dr Ruth Kipping, or one of the members of the NAP SACC UK study team on 0117 9287308 or email napsacc-uk@bristol.ac.uk. They will be happy to assist with any queries.

What to do next

If you are happy to take part in a focus group please complete the reply form on the next page, ticking all relevant boxes and filling in the information requested. Return the form in the prepaid envelope enclosed. One of the research team will then contact you with the time and venue.

If you do not wish to take part in the focus group you do not need to do anything, however you may receive a reminder letter in a few weeks. If you do not wish to take part please ignore this letter.

‘NAP SACC’ study
EARLY YEARS’ AND PUBLIC HEALTH STAFF FOCUS GROUP REPLY AND CONSENT FORM

Name of lead researcher: Dr Ruth Kipping

Please complete all details and return in prepaid envelope:

Please put your initials in box for each question

I do wish to take part in the focus group and I am happy for the NAP SACC UK team to contact me about a time for the focus group	Yes	☐
The purpose of the focus group has been explained to me, and I have been given the chance to discuss any questions or concerns with the researcher.	Yes	☐
I understand that the focus group will be audio-taped but that my responses will remain confidential and I will not be able to be identified in any results.	Yes	☐
I agree to take part in the focus group and understand that I may stop the interview at any time without giving a reason.	Yes	☐

Your full name…………..................……..……………………………………………………….................

Job title …………………………………………………...

Name of organisation you work for ………………………………………………...............................

Address…………………………………………………..

…………………………………………………..

Email. …………………………………………………...

Telephone number...

Preferred time for focus group: Morning ☐ Afternoon ☐ Evening ☐

Signature .... Date …………………………....

Please return form in prepaid envelope provided

NAP SACC UK, University of Bristol, Canynge Hall, 39 Whatley Rd, Bristol BS8 2PS

To be completed by researcher

Researcher name ....

Signature .... Date ………………………….....

'NAP SACC UK Research Study'
The Nutrition and Physical Activity Self Assessment for Child Care

Name of researcher: Dr Ruth Kipping

We are doing some exciting research with nurseries and other child care providers to help them provide young children with healthy food and drink and offer opportunity to do enough exercise. As part of this research we are inviting Health Visitors and Wales Healthy and Sustainable Preschool Scheme staff to help the study by taking part in a focus group.

Would you be willing to take part?

INFORMATION FOR CONSENT FOR FOCUS GROUP

Purpose

It is important for children to do some exercise and eat fruit and vegetables every day for healthy growth and development. Lots of children in England and Wales do not do enough exercise or eat enough fruit and vegetables. One in five children who start primary school are overweight or obese.

This study will aim to test whether we can adapt and use a 6 month programme from the United States called "The Nutrition and Physical Activity Self Assessment for Child Care" (NAP SACC). We will make changes to use it in the UK and test whether we can work with nurseries and other child care providers to make them healthier places for children.

We are very keen to talk to Health Visitors and Wales Healthy and Sustainable Preschool Scheme staff to gain your opinions and find out what you think. We will be asking about changes which could be made so children have more opportunities to have healthy levels of exercise and a healthy diet. As part of this research, you are being invited to participate in a focus group, like a discussion group, with other staff and a researcher. The focus group will take no more than 1.5 hours and will take place in Clevedon for staff working in North Somerset, or Cardiff for staff working in Wales. The information we gather will help us understand the thoughts, opinions and attitudes of health staff working with preschool children, such as yourself. The information we collect will be used to help us make changes to 'NAP SACC' before we trial it in nurseries and other child care providers.

Participation

Your participation in the focus group is voluntary. You can choose not to take part, or you may withdraw at any time. We will pay for your travel expenses to attend the focus group.

Risks

There are no risks associated with taking part in the focus group.

Benefits

The information will help us decide what changes we need to make to the programme and the research study.

Confidentiality

Everything you say during the focus group will remain confidential. No names or identifying information will be used in any results, publication or presentations. The interview session will be audio-taped. Direct quotes (things you have said) from the conversation may be used in reports, but no identifying information will be included (i.e. we will **not** include your name, names of nurseries or other child care providers).

Questions

If you have any further questions about the study, please contact the Principal Investigator, Dr Ruth Kipping, or one of the members of the NAP SACC UK study team on 0117 9287308 or email napsacc-uk@bristol.ac.uk. They will be happy to assist with any queries.

What to do next

If you are happy to take part in a focus group please complete the reply form on the next page, ticking all relevant boxes and filling in the information requested. Return the form in the prepaid envelope enclosed. One of the research team will then contact you with the time and venue.

If you do not wish to take part in the focus group you do not need to do anything, however you may receive a reminder letter in a few weeks. If you do not wish to take part please ignore this letter.

‘NAP SACC’ study
HEALTH VISITORS AND WALES HEALTHY AND SUSTAINABLE PRESCHOOL SCHEME STAFF

FOCUS GROUP REPLY AND CONSENT FORM

Name of lead researcher: Dr Ruth Kipping

Please complete all details and return in prepaid envelope:

Please put your initials in box for each question

I do wish to take part in the focus group and I am happy for the NAP SACC UK team to contact me about a time for the focus group.	Yes ☐
The purpose of the focus group has been explained to me, and I have been given the chance to discuss any questions or concerns with the researcher.	Yes ☐
I understand that the focus group will be audio-taped but that my responses will remain confidential and I will not be able to be identified in any results.	Yes ☐
I agree to take part in the focus group and understand that I may stop the interview at any time without giving a reason.	Yes ☐

Your full name……………...............……...………………………………………………….................

Job title ………………………………………………...

Name of organisation you work for ……………………………………………….................................

Address………………………………………………..

………………………………………………..

Email. ………………………………………………...

Telephone number...

Preferred time for focus group: Morning ☐ Afternoon ☐ Evening ☐

Signature .... Date …………………………....

Please return form in prepaid envelope provided

NAP SACC UK, University of Bristol, Canynge Hall, 39 Whatley Rd, Bristol BS8 2PS

To be completed by researcher

Researcher name ....

Signature .... Date ………………………….....

‘NAP SACC UK Research Study’
The Nutrition and Physical Activity Self Assessment for Child Care

Name of researcher: Dr Ruth Kipping

We are doing some research with nurseries to help them provide young children with healthy food and drink and offer opportunity to do enough exercise. As part of this research we are inviting parents to help the study by taking part in a interview.

Would you be willing to take part?

INFORMATION FOR CONSENT FOR PARENT INTERVIEW

What is the purpose of the research?

It is important for children to do some exercise and eat fruit and vegetables every day for healthy growth and development. Lots of children in England and Wales do not do enough exercise or eat enough fruit and vegetables. One in five children who start primary school are overweight or obese.

This study will aim to test whether we can adapt and use a 6 month programme from the United States called “The Nutrition and Physical Activity Self Assessment for Child Care” (NAP SACC). We will make changes to use it in the UK and test whether we can work with nurseries and other child care providers to make them healthier places for children.

We are very keen to gain parents’ opinions and find out what you think. In 2015 we undertook interviews with parents and we are contacting these parents again to ask you to take part in an interview. We will be telling you about the way we now aim to involve parents using a website, emails, text messages and Facebook. We would like to ask for your comment about how to involve parents and for your comment on the materials we plan to use.

The interview will take no more than 60 minutes and will take place over the telephone. We will arrange to phone you at a time which is convenient to you. The information we gather will help us understand the thoughts, opinions and attitudes of parents with children at nursery, such as yourself. The information we collect will be used to help us make changes to ‘NAP SACC’ before we trial it in nurseries.

Who is taking part?

If you took part in the telephone interview in 2015 you can take part in this study. Your participation in the interview is voluntary. You can choose not to take part, or you may withdraw at any time. If you choose to participate you will be given a £10 shopping voucher (‘high street’ voucher which is suitable in many supermarkets and high street shops) to thank you for your time.

If you indicate you would like to take part in the interview, the researcher will contact you to confirm the date and time.

What are the risks?

There are no risks associated with taking part in the interview. As said above, you will be compensated for your time with a £10 shopping voucher.

What are the benefits?

Information collected from the interview will provide a better understanding of how to involve parents and further changes we need to make to the NAP SACC at Home intervention.

Will the information be confidential?

Everything you say during the interview will remain confidential. No information will be given to your nursery. No names or identifying information will be used in any results, publication or presentations. The interview will be audio-taped. Direct quotes (things you have said) from the conversation may be used in reports, but no identifying information will be included (i.e. we will **not** include your name, your child’s name, or the name of your nursery).

What should I do if I have questions?

If you have any further questions about the study, please contact the Principal Investigator, Dr Ruth Kipping, or one of the members of the NAP SACC UK study team on 0117 9287308 or email napsacc-uk@bristol.ac.uk. They will be happy to assist with any queries.

What should I do next?

If you are happy to take part in the interview, please complete the reply form on the next page, ticking all relevant boxes and filling in the information requested. Return the form in the prepaid envelope enclosed. One of the research team will then contact you to book a time which is convenient for you to do the interview.

If you do not wish to take part in the interview you do not need to do anything, however you may receive a reminder letter in a few weeks. If you do not wish to take part please ignore this letter.

‘NAP SACC’ study
PARENT INTERVIEW REPLY AND CONSENT FORM

Name of lead researcher: Dr Ruth Kipping

Please complete all details and return in prepaid envelope:

Please put your initials in box for each question

I have read and understood the information sheet (version 1; dated 05/11/15)	Yes [ ]
I do wish to take part in the parent interview and I am happy for the NAP SACC team to contact me to inform me of the date, time and venue.	Yes [ ]
The purpose of the interview has been explained to me, and I have been given the chance to discuss any questions or concerns with the researcher.	Yes [ ]
I understand that the interview will be audio-taped but that my responses will remain confidential and I will not be able to be identified in any results.	Yes [ ]
I agree to take part in the interview and understand that I may stop the interview at any time without giving a reason.	Yes [ ]
I understand that the information collected will be used to support other research in the future, and may be shared anonymously with other researchers.	Yes [ ]

Name of parent................……..………………………………………………………..............

Best telephone number to contact (including code) ……………………………….................

Email address: ……………………………………………………………………………………

Best time of day to contact you (please tick) am [ ] pm [ ] evening [ ]

I am available for the interview on the following dates and times:

19 November [ ] Morning [ ] Afternoon

20 November [ ] Morning [ ] Afternoon

25 November [ ] Morning [ ] Afternoon

26 November [ ] Morning [ ] Afternoon

27 November [ ] Morning [ ] Afternoon

Signature .... Date ………………………………...

Please return form in prepaid envelope provided

NAP SACC UK, University of Bristol, Canynge Hall, 39 Whatley Rd, Bristol BS8 2PS

To be completed by researcher

Researcher name ....

Signature .... Date …………………………......

‘NAP SACC UK Research Study’
The Nutrition and Physical Activity Self Assessment for Child Care

Name of researcher: Dr Ruth Kipping

We are doing some exciting research with nurseries and other child care providers to help them provide young children with healthy food and drink and offer opportunity to do enough exercise. As part of this research we are inviting parents to help the study by taking part in a telephone interview.

Would you be willing to take part?

INFORMATION FOR CONSENT FOR PARENT INTERVIEW

Purpose

It is important for children to do some exercise and eat fruit and vegetables every day for healthy growth and development. Lots of children in England and Wales do not do enough exercise or eat enough fruit and vegetables. One in five children who start primary school are overweight or obese.

This study will aim to test whether we can adapt and use a 6 month programme from the United States called “The Nutrition and Physical Activity Self Assessment for Child Care” (NAP SACC). We will make changes to use it in the UK and test whether we can work with nurseries and other child care providers to make them healthier places for children.

We are very keen to gain parents’ opinions and find out what you think. We will be asking about changes which could be made so children have more opportunities to have healthy levels of exercise and a healthy diet. As part of this research, you are being invited to participate in an interview with one of researchers. The interview will take no more than 60 minutes and will take place over the telephone. The information we gather will help us understand the thoughts, opinions and attitudes of parents with children aged 2-4, such as yourself. The information we collect will be used to help us make changes to ‘NAP SACC’ before we trial it in nurseries and other child care providers.

Participation

If you have a child who is aged 2-4 who spends time at a nursery or child care provider you can take part in the study. Your participation in the interview is voluntary. You can choose not to take part, or you may withdraw at any time. If you choose to participate you will be given a £10 shopping voucher (‘high street’ voucher which is suitable in many supermarkets and high street shops) to thank you for your time.

If you take part in a telephone interview, the researcher will arrange to call you at a time that is convenient to you. The phone call will be made from the researcher, so you will not be charged for the call on your telephone bill.

Risks

There are no risks associated with taking part in the interview. As said above, you will be compensated for your time with a £10 shopping voucher.

Benefits

Information collected from the interviews will provide a better understanding of knowledge and parents’ attitudes about healthy eating, drinking, exercise and sedentary behaviour (inactive behaviour such as sitting and watching TV/playing computer games). The information will help us decide what changes we need to make to the programme and the research study.

Confidentiality

Everything you say during the interview will remain confidential. No information will be given to your nurseries and other child care provider. No names or identifying information will be used in any results, publication or presentations. The interview session will be audio-taped. Direct quotes (things you have said) from the conversation may be used in reports, but no identifying information will be included (i.e. we will **not** include your name, your child’s name, or the name of your nurseries and other child care provider).

Questions

If you have any further questions about the study, please contact the Principal Investigator, Dr Ruth Kipping, or one of the members of the NAP SACC UK study team on 0117 9287308 or email napsacc-uk@bristol.ac.uk. They will be happy to assist with any queries.

What to do next

If you are happy to take part in an interview, please complete the reply form on the next page, ticking all relevant boxes and filling in the information requested. Return the form in the prepaid envelope enclosed. One of the research team will then contact you to book a time which is convenient for you to do the interview.

If you do not wish to take part in the interviews you do not need to do anything, however you may receive a reminder letter in a few weeks. If you do not wish to take part please ignore this letter.

‘NAP SACC’ study

PARENT INTERVIEW REPLY AND CONSENT FORM

Name of lead researcher: Dr Ruth Kipping

Please complete all details and return in prepaid envelope:

Please put your initials in box for each question

I do wish to take part in the parent interviews am happy for the NAP SACC to telephone me to arrange a convenient time for the interview	Yes [ ]
The purpose of the interview has been explained to me, and I have been given the chance to discuss any questions or concerns with the researcher.	Yes [ ]
I understand that the interview will be audio-taped but that my responses will remain confidential and I will not be able to be identified in any results.	Yes [ ]
I agree to take part in the interview and understand that I may stop the interview at any time without giving a reason.	Yes [ ]

Name of parent.................……..………………………………………………………….......

Age of child at nursery/child care provider …………………………………………….....

Name of nursery/child care provider ………………………………………….....................

Best telephone number to contact (including code) ………………………………….................

Best time of day to contact you (please tick) am [ ] pm [ ] evening [ ]

Signature .... Date ………………………………..

Please return form in prepaid envelope provided

NAP SACC UK, University of Bristol, Canynge Hall, 39 Whatley Rd, Bristol BS8 2PS

To be completed by researcher

Researcher name ....

Signature .... Date …………………………….....

Contact: Ruth Glover

Direct dial: 01275 884 389

Email: ruth.glover@n-somerset.gov.uk

Early Years Team

People and Communities – CYPS

North Somerset Council

Post Point 19

Town Hall

Date xx/xx/xx

Dear Nursery Manager

Invitation to take part in 'NAPSACC UK' Research Study

I am writing to invite your nursery to take part in a new research study taking place in nurseries from September 2015-October 2016. The study is being run by the University of Bristol and will aim to test whether they can adapt and use a programme from the United States called "The Nutrition and Physical Activity Self Assessment for Child Care" (NAP SACC). They have made some changes in order to use it in the UK and are testing whether they can work with nurseries to make their environment healthier.

Please read the attached information sheet entitled ('*NAP SACC UK Research Study*') which explains what participation in this study will entail. Someone from the study team will contact you by telephone within the next week to arrange a time to visit your nursery to explain the study fully and give you the opportunity to ask any questions before you decide whether to proceed. Alternatively, if having read the attached information you know whether you would like to participate, you can complete and return the attached **reply form**.

If you have any questions about the study in the meantime, please contact the Principal Investigator, Dr Ruth Kipping, or one of the members of the NAP SACC UK study team at the University of Bristol on 0117 928 7308. They will be happy to assist with any queries.

Yours sincerely,

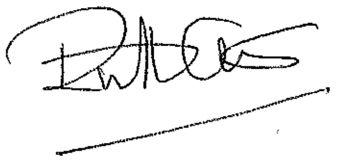

Ruth Glover

Interim Head of Early Years

‘NAP SACC UK Research Study’
The Nutrition and Physical Activity Self Assessment for Child Care

What is the study?

It is important for children to do some exercise and eat fruit and vegetables every day for healthy growth and development. Lots of pre-school-aged children in England and Wales do not do enough exercise or eat enough fruit and vegetables. One in five children who start primary school are overweight or obese.

This study tests whether we can adapt and use a 6 month programme from the United States called “The Nutrition and Physical Activity Self Assessment for Child Care” (NAP SACC). We have made some changes in order to use it in the UK and will test whether we can work with nurseries to make them healthier places for children.

NAP SACC UK is an intervention delivered in nursery settings with the aim of improving policies, practices and the nutrition and physical activity environment, through a process of review and targeted assistance from ‘NAP SACC UK Partner’ (a health visitor). The steps of the intervention are as follows:

*1. **Review and Reflect:*** The nursery manager, together with key nursery staff complete the NAP SACC UK ‘Review and Reflect’ tool. This tool assesses the nursery on 15 key areas in nutrition and physical activity with response options ranging from minimal to best practice.

*2. **Action Planning**:* Based on the ‘Review and Reflect’ answers, nurseries will choose areas for improvement and set goals with guidance and support from a NAPSACC UK Partner.

*3. **Workshop Delivery**:* The trained NAP SACC UK Partner delivers a workshop to the staff. The workshop will cover nutrition and physical activity for children and will engage all the nursery staff in action planning to meet the goals set by the nursery manager and NAP SACC UK partner.

*4. **Targeted technical assistance:*** NAP SACC UK Partners maintain regular contact with the nursery to provide support and guidance in making their improvements.

5. ***Evaluate, Revise, and Repeat:*** The NAP SACC 'Review and Reflect' tool is completed a second time to see where improvements have or haven't been made. At this time Action Plans are revised to include new goals and objectives and technical assistance continues.

What about involving parents?

We are designing a new NAP SACC UK at home component, to encourage parents and carers to make healthy changes at home. This is under development.

Who is participating in the study?

Twelve nurseries will participate in the trial, of which 6 will be randomised to the control group and 6 to the intervention. The nurseries in the control group will continue with their usual planned activities and policies. There will be no placebo intervention for the control group.

The data will be collected from 2-4 year old children who attend the nursery for at least 12 hours per week over 50 weeks of the year or 15 hours per week in term time, who are provided with lunch (or another main meal) every day. Participating nurseries must have at least 20 eligible children attending.

How will nurseries be allocated to 'intervention' or 'control' nurseries?

A computer programme will be used to randomly allocate nurseries to intervention or control nurseries. This is important because to test whether the intervention works we need to have a 'fair' comparison between the nurseries getting the intervention and the control nurseries. To do this we need to make sure that those who get the intervention are chosen by chance (luck) rather than by any particular characteristics.

What do nursery staff have to do?

Nurseries taking part in the study are asked to commit to the following:

- To send out letters and information sheets, provided by the project team, to parents. These will give parents information about the study and ask if the parents consent to their child having the measurements taken. The letters will be signed by Dr Ruth Kipping (the project lead from the University of Bristol).

- To allow a meeting between parents and research staff to take place at the nursery, so parents can find out more about the study.

- To provide time for a researcher (with enhanced DBS check) from the University of Bristol to come to the nursery to do the measurements with the participating 2-4 year olds. This will require a nursery carer to be present during the height and weight measurements.

- To give out a questionnaire for parents to complete about their child's dietary intake (provided by the study team).

- To take part in the five aspects of NAP SACC UK outlined on the previous page, if the nursery is an 'intervention' nursery and to send home resources for the 'NAP SACC UK at Home' component of the study.

- To provide a member of staff to do an interview to give feedback at the end of the study.

What do parents have to do?

- Parents/carers will be sent a letter, project information sheet, reply envelope and form indicating if they wish to give consent for their child to take part in the data collection.

- At baseline (prior to starting the intervention) and follow-up (1 year later) parents will be asked to complete a questionnaire about their child's dietary intake; non-returns will be contacted by telephone by a field worker. Parents will also be asked to complete a short questionnaire, 'About You and Your Preschool Child'.

- Parents whose children are in 'intervention' nurseries will be given resources as part of the 'NAP SACC UK at Home' part of the intervention.

What do children have to do?

- At the baseline and follow-up data collections, the child's weight and height will be measured by a trained researcher in the nursery, with a member of nursery staff present. The children will be fitted with ActiGraph accelerometers (which look like pedometers) to calculate physical activity and sedentary behaviours.

- Child care staff and parents will be given simple instructions and advice about the child wearing the accelerometer.

How will the measurements be done?

- All measurements will be collected by University staff with enhanced DBS clearance. These staff will be trained and experienced at collecting information and measurements from children.

- The height and weight measurements will be done in a private area away from other children and staff. Children who take part will be asked to remove their shoes and any heavy outdoor clothing. They will be weighed in normal indoor clothing. Staff and other children at the nursery will not see the measurements. However, to give reassurance to the children and for child protection, a member of nursery staff will be present.

- The university staff will explain how the children should wear the accelerometers and will collect these from the nurseries at the end of the 6 days of wearing them.

Risks

The risks associated with taking part in the study are minimal. A potential risk is that that some children or parents might find some aspects of the study upsetting if they are particularly sensitive to issues of food or weight. The Principal Investigator (Dr Ruth Kipping) will work with your nursery to ensure a system is in place to enable appropriate support in such circumstances. We will minimise children being upset about having their weight measured by a member of nursery staff being present during the measurements.

NAP SACC UK aims to promote physical activity in children. Therefore there is a small risk of increased injuries relating to usual children's physical activity. However we do not anticipate more risky activities than would usually take place at nursery or outside the nursery.

Benefits

There are potential benefits to your nursery in taking part in this research.

- Participation can be cited as evidence to Ofsted of contribution to children's wellbeing and effectiveness of leadership and management during the Ofsted inspection of early years' providers.
- You will have direct contact with a named health visitor for a 6 month period
- You will receive a visit from a Physical Activity Specialist to help advise you on making the most of your indoor and outdoor space and give you ideas to keep children active
- There are potential benefits for children taking part with regard to changes being made to the nursery and home environment which could benefit their health.

Confidentiality

All information you provide us with during the study will remain confidential. No information will be passed on to members of your nursery staff, parents or other nurseries. No names or identifying information will be used in any results, publication or presentations. However, if anything is disclosed or observed where there is serious concern about the health or well-being of a child, either the nursery manager will be informed or the information will be shared with an appropriate organisation.

Who is leading the project?

The project is being led by Dr Ruth Kipping, University of Bristol, with guidance from staff from North Somerset Council, Bristol Council, Gloucestershire County Council and experts in child nutrition and physical activity from the University of Bristol, University of Cardiff, University of Glasgow, University College London, University of Exeter, and University of Southampton.

Ethical approval

This project has been reviewed and approved by a National Health Service (NHS) Research Ethics Committee (REC); Wales REC 3.

Who is funding the study?

The University of Bristol has been funded by the National Institute for Health Research to conduct the NAPSACC UK Study.

What are the timescales?

The intervention will take place over 6 months. The baseline data collection will take place prior to the intervention. Follow-up assessment will take place 9-12 months after the baseline data collection.

Reimbursement for time

All participating nurseries will receive **£200**, whether intervention or control, at the end of the study.

What do I need to do next?

Someone from the NAP SACC UK study team will contact you by telephone within the next week to arrange a convenient time for a visit. At the visit we will explain the study to you and give you the opportunity to ask any questions before you decide whether you would like to participate.

If you already know whether you would like to participate, you canfill out the attached **reply form** and return it, at your earliest convenience, in the prepaid envelope enclosed. In this case, if you are interested, we will arrange a meeting or a telephone call to talk to you further about the study. After that we will ask you to give your agreement (consent) to take part if you would still like to be involved.

What if I change my mind?

Your nursery's participation in the study is voluntary. You can choose not to take part, or you may withdraw your nursery at any time.

Questions

If you have any further questions about the study, please contact the Principal Investigator, Dr Ruth Kipping, or one of the members of the NAP SACC UK study team on 0117 9287308 or email napsacc-uk@bristol.ac.uk

‘NAP SACC’ UK STUDY
NURSERY

REPLY FORM

Name of Principal Investigator: Dr Ruth Kipping

Name of member of staff...………………………………………………………..…...

Job Title...……………………………………………………………………..………...

Name of nursery…………………………..…………………………………………..…..

Address……………………….……………………………………………………..…

……………………….……………………………………………………………………….

Contact telephone number..………………………………………………………………

Email address…………………………………………….....…………………………

A. I am interested in taking part in the above study ☐ *(tick)*

i) Number of children aged 2 – 4, who will be attending for at least 12 hours per week (or 15 hours term time only) from Sept 2015 ……………………………………….....

ii) Is a main meal provided each day (this does not include packed lunch)? Yes [ ] No [ ]

OR

B. I am not interested in taking part in the above study ☐ *(tick)*

For research purposes it is very helpful if you can give a brief reason: ..…...…………
…………………………………………………………………………………………………

Signature …………………………………………………… Date…………...………

Please return this form at your earliest convenience, in the addressed **prepaid envelope provided**.

If envelope has been mislaid please post to:

NAP SACC UK, University of Bristol, 4.09 Canynge Hall, 39 Whatley Rd, Bristol BS8 2PS

‘NAP SACC’ STUDY
NURSERY

CONSENT FORM

Name of Principal Investigator: Dr Ruth Kipping

Please put your initials in the box for each question

I do wish for my nursery to take part in the study and I am happy for the NAP SACC UK team to contact me about a date and time for data collection.	Yes	☐
The purpose of the study has been explained to me, and I have been given the chance to discuss any questions or concerns with the researcher.	Yes	☐
I understand that all information nursery staff provide will remain confidential and nursery staff will not be able to be identified in any results.	Yes	☐
I understand that my nursery may stop participating at any time without giving a reason.	Yes	☐
I understand that the information collected will be used to support other research in the future, and may be shared anonymously with other researchers.	Yes	☐

Name of member of staff............…...………………………………………………….................

Job title ………………………………………………...

Name of nursery …………………………………………….................................

Telephone number..

Email address...

Signature .... Date ………………………………

Please return form in prepaid envelope provided

NAP SACC UK, University of Bristol, Canynge Hall, 39 Whatley Rd, Bristol BS8 2PS

To be completed by researcher

Researcher name ....

Signature .... Date …………………………….....

SCHOOL OF SOCIAL AND COMMUNITY MEDICINE
Canynge Hall, Whately Road, Bristol, BS8 2PS

Dr Ruth Kipping

T +44 (0)117 928 7273

ruth.kipping@bristol.ac.uk

http://www.epi.bris.ac.uk

Date: xx/xx/xx

Dear Parent/Carer

Invitation for your child to take part in a University of Bristol Research Study

Your child’s nursery is taking part in a new research study taking place in nurseries in North Somerset and Gloucestershire from September 2015-October 2016. This research will look at how we can make changes to the nursery and home environments to help pre-school-aged children to do more exercise and eat a healthy diet.

Please take your time to read the attached information sheet entitled (‘*NAP SACC UK Research Study*’) which explains what taking part in this study will involve. If you are happy for your child to participate please **complete the attached CONSENT FORM and brief questionnaire**. We would be grateful if you could return these forms in the addressed prepaid envelope which has been included for your convenience.

If you have any further questions about the study, you can contact the Principal Investigator, Dr Ruth Kipping, or one of the members of the NAP SACC UK study team on 0117 928 7308. We will be happy to assist with any queries.

Yours sincerely,

Dr Ruth Kipping

Research Fellow

‘NAP SACC UK Research Study’
The Nutrition and Physical Activity Self Assessment for Child Care

PARENT INFORMATION SHEET

We plan to do some research in local nurseries to look at how we make changes to the nursery and home environments to help pre-school-aged children to do more exercise and eat a healthy diet. As part of this research we would like you to fill in a short questionnaire entitled ‘About You and Your Preschool Child’ and another questionnaire about your child’s dietary intake. We will also ask if you are happy for your child to wear a belt around their waist with a small box that measures how active they are and for your child to be weighed and measured.

Before you decide whether or not you would like your child to take part, it is important you understand why the research is being done and what it will involve. Please take time to read and consider the following information carefully. You may wish to discuss the information with a friend or family member to help you decide. Please ask us if anything is unclear or you have any further questions.

What is the purpose of the study?

- Doing some exercise and eating fruit and vegetables every day is important for healthy growth and development in children.
- Lots of pre-school children in England and Wales do not do enough exercise or eat enough fruit and vegetables.
- This study tests whether we can adapt and use a 6 month programme from the United States called “The Nutrition and Physical Activity Self Assessment for Child Care” (NAP SACC). We have made some changes in order to use it in the UK and will test whether we can work with nurseries to make them healthier places for children.

Which nurseries and which children are in the study?

- 12 nurseries (6 in North Somerset and 6 in Gloucestershire) will be participating in this study
- Your child's nursery is taking part in the study
- We are inviting all 2-4 year old children who attend the nursery for at least 12 hours per week over 50 weeks of the year or 15 hours per week in term time, who are provided with lunch (or another main meal), to take part
- Children are not chosen because of their weight or for any other reason

What will be measured?

We are asking parents/carers if they would be happy to give consent (agreement) for their child to take part in the following measurements on two occasions (one year apart).

We would like to measure your child's:

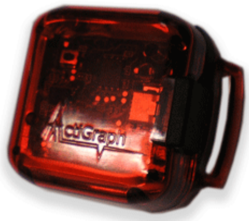

- Activity level. We will measure this by asking them to wear an 'accelerometer' for 6 days. This is a small activity monitor which look like a pedometer (see picture) and is worn on a belt around your child's waist during day-time.
- Height and weight
- Dietary Intake. We are asking parents if they would be willing to fill in a questionnaire about their child's dietary intake (on two occasions, approximately one year apart) and another questionnaire entitled 'About You and Your Preschool Child' (enclosed with this letter).

How will the measurements be done?

- All measurements will be collected by University staff with enhanced 'Disclosure and Barring Service' (DBS) clearance. These staff will be trained and experienced at collecting these measurements from children.
- The height and weight measurements will be done in a private area away from other children. To make the children feel comfortable one member of nursery staff will be with the child during the measurements. Staff and other children at the nursery will not see the measurements. Children who take part will be asked to remove their shoes and any heavy outdoor clothing. They will be weighed in normal indoor clothing.
- If a child makes it clear that they do not want to take part in the measurements, the measurements will not be taken, even if their parent/carer has given agreement for them to participate. Instead they will carry on with normal nursery activities.
- If you would like to be present while your child is being measured, please let us know and we will be able to give you the date and time of the measurement session.
- The university staff will explain how the children should wear the accelerometers and will collect these from the nurseries at the end of the 6 days of wearing them.

What happens to the measurements?

- The information collected about your child will be kept confidential and will only be used for research.
- We will not be providing parents with their child's Body Mass Index (BMI) or providing feedback on whether their child is underweight, normal weight or overweight. Please note that this study is not part of the National Child Measurement Programme

(NCMP) / Child Measurement Programme for Wales, which children take part in during Reception Class at Primary School.

- The information will be stored at the University of Bristol.
- All questionnaires and computer data for your child will be stored with an ID code (rather than their name) and will be anonymised so that it will not be possible for anyone to see information connected with your child.
- When the data is analysed the information will be anonymous, so that it will not be possible to identify an individual child.
- None of your child's information will be passed on to the nursery or any other individual or organization who is not directly involved with analysing the data for the study research. Those analysing the data for research purposes will not have your child's name or personal details. In short, no one will be able to find out your child's measurements. However, if anything is disclosed or observed where there is serious concern about the health or well-being of a child, either the nursery manager will be informed or the information will be shared with an appropriate organisation.
- In the future, the data collected may be used in other studies. For example, future researchers may wish to compare the diet of children in the NAP SACC study, with the diet of children in a different study. However, it would not be possible to identify your child in any way. The new researchers would not know your child's name, address, nursery name, or any other identifiable information about them.

Who is leading the project?

The project is being led by Dr Ruth Kipping, University of Bristol, with guidance from staff from North Somerset Council, Bristol Council and Gloucestershire County Council and experts in child nutrition and physical activity from the University of Bristol, University of Cardiff, University of Glasgow, University College London, University of Exeter, and University of Southampton.

Ethical approval

This project has been reviewed and approved by a National Health Service (NHS) Research Ethics Committee (REC); Wales REC 3.

Who is funding the study?

The University of Bristol has been funded by the National Institute for Health Research to conduct the NAPSACC UK Study.

Reimbursement for time

All participating children will receive a small toy and certificate to thank them for their participation in the study.

What do I need to do next?

If you are happy for your child to take part in the study please fill out the attached **consent form and brief questionnaire**, and return it **as soon as possible** (using the pre-paid

addressed envelope) to the NAPSACC UK Study team. Please note there is no obligation for your child to take part in the study.

What if I change my mind?

You are free to withdraw consent for your child to take part in the study at any stage without needing to give a reason. Please contact the research team at any time if you change your mind. If you decide that you no longer want your child's data to be included in the study, it can be deleted up to the point of analysis. After the analysis stage we are unable to remove an individual child's data.

Questions

If you wish to know more about the study before deciding whether to take part, or if at a later date you want to withdraw from the study please telephone the NAPSACC UK Research Team on 0117 9287308 or email napsacc-uk@bristol.ac.uk.

NAP SACC UK STUDY: PARENT CONSENT FORM

Name of Principal Investigator: Dr Ruth Kipping

Child's name: ………………………………………………………………………………

Child's date of birth: ……………………………………………………………………

Child's nursery: …………………………………………………………………………

*Please enter your **initials** in the box to indicate you agree with the statement*

I have read and understood the parent information sheet	Yes ☐
I have been given contact details of the research team if I wish to discuss any questions or concerns	Yes ☐
I give consent for my child to participate in the NAP SACC UK study and to participate in the data collection and measurements as outlined in the parent information sheet	Yes ☐
I understand that all data will remain confidential and my child will not be able to be identified in any results.	Yes ☐
I understand that I can withdraw my permission for my child to participate at any time without giving a reason.	Yes ☐
I understand that the information collected will be used to support other research in the future, and may be shared anonymously with other researchers but my child will not be able to be identified in any results.	Yes ☐

Parent's / Carer's name: ……………………………………………………………….…………

Parent's / Carer's signature: ……………………………………………Date….…………..

Please return this form, **within the next 7 days**, in the addressed **prepaid envelope provided,** along with the **questionnaire entitled 'About You and Your Child'.**

If envelope has been mislaid please post to:

NAP SACC UK, University of Bristol, 4.09 Canynge Hall, 39 Whatley Rd, Bristol BS8 2PS

To be completed by researcher

Researcher name ....

Signature .... Date …………………………......

NAP SACC UK Study

About You and Your Preschool Child

To be filled in by the child's primary caregiver

As part of our research we would like to know a little bit about you and the 2-4 year old child in your care.

Q1. Child's First Name:______________________ **Q2. Child's Last Name:** ______________________

Q3. Name of Child's Nursery____

Q4. Your First Name:_____________________ **Q5. Your Last Name:** ____________________

	Mother	Father	Step-Parent	Carer/ Guardian	Grand-parent	Other Relative
**Q6. What is your relationship to your 2-4 year old child?**	☐	☐	☐	☐	☐	☐

Q7. Child's Gender: Male ☐ **Female** ☐

Q8. a) YOUR date of birth: ___/___/___ **b) CHILD's date of birth: ___/___/___**

Q9. How would you describe <u>your</u> ethnic origin?

- ☐ White British
- ☐ White Other
- ☐ Black British
- ☐ Black Caribbean
- ☐ Black African
- ☐ Asian British
- ☐ Indian
- ☐ Pakistani
- ☐ Bangladeshi
- ☐ Chinese
- ☐ Mixed (please write below) ____________________________
- ☐ Other (please write below) ____________________________
- ☐ I would rather not answer

Q10. How would you describe <u>your child's</u> ethnic origin?

- ☐ White British
- ☐ White Other
- ☐ Black British
- ☐ Black Caribbean
- ☐ Asian British
- ☐ Indian
- ☐ Pakistani
- ☐ Bangladeshi
- ☐ Mixed (please write below) ____________________________
- ☐ Other (please write below) ____________________________

☐ Black African ☐ Chinese ☐ I would rather not answer

Please select **ONE BOX** for **EACH** question:

	Up to GCSEs/GCEs/O levels or similar	A levels/NVQs/ GNVQs	First degree/ diploma/HNC/ HND	Higher degree (e.g. MSc, PhD)
**Q11. What is the highest level of education you have achieved?**	☐	☐	☐	☐

	1	2	3	4	5	6 or more
**Q12. How many children are in your household (including your 2-4 year old)?**	☐	☐	☐	☐	☐	☐

	Mother	Father	Step-Parent	Carer/ Guardian	Grand-Parent	Other Relative
**Q13. Who usually lives in the same home as your child on a WEEKDAY? (tick all that apply)**	☐	☐	☐	☐	☐	☐

	Mother	Father	Step-Parent	Carer/ Guardian	Grand-Parent	Other Relative
**Q14. Who usually lives in the same home as your child on a WEEKEND? (tick all that apply)**	☐	☐	☐	☐	☐	☐

Q15. Days and hours child will attend this nursery from September 2015 (do not include time spent at other nurseries):

Monday Yes [ ] / No [ ] If Yes, please complete time: From________ until ________

Tuesday Yes [ ] / No [ ] If Yes, please complete time: From________ until ________

Wednesday Yes [ ] / No [ ] If Yes, please complete time: From________ until ________

Thursday Yes [ ] / No [ ] If Yes, please complete time: From________ until ________

Friday Yes [ ] / No [ ] If Yes, please complete time: From________ until ________

Q16. Name of room/class your child will be in from September 2015 (if known):

Room/class name: ___________________________ / Not known [ ] / NA (i.e. 1 room only) [ ]

Q17. We will need to contact you via post, telephone and email in the future to complete data collection and to give you information about the study. Please provide the following:

Home Address: ____

____

____Postcode:_____________________________

Tel number:____

Email address:____

Thank you for completing this questionnaire.

Please return along with the consent form, in the prepaid envelope provided

If English is not your first language and you need a translation, we can get one for you. Contact: Sian Wells Telephone: 0117 928 7308

ALBANIAN

Nëse anglishtja nuk është gjuha juaj amtare dhe keni nevojë për një përkthim, ne mund t'ua sigurojmë atë.

BENGALI

ইংরেজী আপনার মাতৃভাষা না হলে এবং আপনার কোন অনুবাদের প্রয়োজন হলে আমরা তা প্রদান করতে সক্ষম।

CHINESE

如果英文不是您的第一語言，而您需要翻譯的話，我們可以爲您安排。

GUJARATI

જો તમારી પહેલી ભાષા અંગ્રેજી ન હોય અને તમને ભાષાંતરની જરૂર હોય તો અમે તમને તે આપી શકીએ છીએ.

HINDI

यदि अँग्रेज़ी आप की पहली भाषा नहीं है और आप को अनुवाद की आवश्यकता है तो यह हम आपको प्रदान कर सकते हैं

KURDISH

Heke îngilîzî zimanê we yê yekem nîne û pêwîstîya we bi wergêr heye, em dikarin yekî ji we re bibînin

POLISH

Jeżeli język angielski nie jest Twoim językiem ojczystym i wymagasz tłumaczenia, możemy to zapewnić.

PORTUGUESE

Se o Inglês não é a sua língua materna e precisa de uma tradução, nós podemos obtê-la.

PUNJABI

ਜੇਕਰ ਇੰਗਲਿਸ਼ ਤੁਹਾਡੀ ਪਹਿਲੀ ਭਾਸ਼ਾ ਨਹੀਂ ਅਤੇ ਤੁਹਾਨੂੰ ਦੁਭਾਸ਼ੀਏ ਦੀ ਜ਼ਰੂਰਤ ਹੈ ਤਾਂ ਤੁਹਾਡੇ ਲਈ ਅਸੀਂ ਇਸਦਾ ਪ੍ਰਬੰਧ ਕਰ ਸਕਦੇ ਹਾਂ ।

SOMALI

Haddii Ingiriisku aanu ahayn afkaaga kowaad oo aad u baahan tahay turjumaad, annagaa kuu samayn karra.

URDU

اگر انگریزی آپ کی پہلی زبان نہیں ہے اور آپ کو ترجمہ کی ضرورت ہے تو ہم آپ کے لئے فراہم کر سکتے ہیں۔

VIETNAMESE

Nếu quí vị không thao Anh văn và cần bản dịch, chúng tôi sẽ giúp quí vị một bản.

‘NAP SACC UK Research Study’
The Nutrition and Physical Activity Self Assessment for Child Care

Name of researcher: Dr Ruth Kipping

We are doing some research with nurseries to improve the health of young children. As part of this research we are inviting Managers of Nurseries which have taken part in the NAP SACC UK study to take part in an interview.

Would you be willing to take part?

INFORMATION FOR NURSERY MANAGER’S CONSENT FOR INTERVIEW

What is the purpose of the research?

It is important for children to do some exercise and eat fruit and vegetables every day for healthy growth and development. Lots of children in England and Wales do not do enough exercise or eat enough fruit and vegetables. One in five children who start primary school are overweight or obese.

This study will aim to test whether we can adapt and use a 6 month programme from the United States called “The Nutrition and Physical Activity Self Assessment for Child Care” (NAP SACC). We have made changes to use it in the UK and have tested whether we can work with nurseries to make them healthier places for children.

We would like to find out what Nursery Managers think about the NAP SACC UK study. As part of this research, you are being invited to participate in an interview with a researcher. The interview will take no more than 1 hour and will take place at a time which is convenient to you. It will take place over the phone or at your nursery – whichever you prefer. The information we gather will help us understand the thoughts and opinions of Nursery Managers who have been involved in the study. The information we collect will be used to help us decide how to use ‘NAP SACC UK’ in the future.

Who can take part?

Your participation in the interview is voluntary. You can choose not to take part, or you may withdraw at any time.

What are the risks?

There are no risks associated with taking part in the interview.

What are the benefits in taking part?

The information will help us decide what changes we need to make to the programme and the research study.

Will the information I give be confidential?

Everything you say during the interview will remain confidential. No information will be given to your nursery or other child care providers. No names or identifying information will be used in any results, publication or presentations. The interview session will be audio-taped. Direct quotes (things you have said) during the training may be used in reports, but any quotes will be anonymous. No identifying information will be included in quotes so that you cannot be identified (i.e. we will **not** include your name or the name of your employer).

The main circumstances under which the researchers would break confidentiality are where an individual is regarded to be at risk of serious harm. All participants will be informed that if they disclose information about neglect or abuse we will pass this information on to an appropriate source.

What do I do if I have questions?

If you have any further questions about the study, please contact the Principal Investigator, Dr Ruth Kipping, or one of the members of the NAP SACC UK study team on 0117 928 7308 or email napsacc-uk@bristol.ac.uk. They will be happy to assist with any queries.

What should I do next?

If you are happy to take part in an interview please complete the reply form on the next page, signing all relevant boxes and filling in the information requested. Return the form in the prepaid envelope enclosed. One of the research team will then contact you with the time and venue.

If you do not wish to take part in the interview you do not need to do anything, however you may receive a reminder letter in a few weeks. If you do not wish to take part please ignore this letter.

‘NAP SACC’ study

NURSERY MANAGER INTERVIEW REPLY & CONSENT FORM

Name of lead researcher: Dr Ruth Kipping

Please complete all details and return in prepaid envelope:

Please put your initials in box for each question

I have read and understood the information sheet (version 1; dated 16/09/15)	☐
I do wish to take part in the interview and I am happy for the NAP SACC UK team to contact me about a time for the interview	☐
The purpose of the interview has been explained to me, and I have been given the chance to discuss any questions or concerns with the researcher.	☐
I understand that the interview will be audio-taped but that my responses will remain confidential. Any use of direct quotes will be anonymised and I will not be able to be identified in any results.	☐
I agree to take part in the interview and understand that I may stop the interview at any time without giving a reason.	☐
I understand that the information collected will be used to support other research in the future, and may be shared anonymously with other researchers.	☐

Name of member of staff...

Job title ....

Name of nursery/child care provider ....

Address...

....

Telephone number...

Preferred time for interview: Morning ☐ Afternoon ☐ Evening ☐

Preferred place for interview: Face to face at Nursery ☐ By telephone ☐

Signature .... Date ………………………………

Please return form in prepaid envelope provided

NAP SACC UK, University of Bristol, Canynge Hall, 39 Whatley Rd, Bristol BS8 2PS

To be completed by researcher

Researcher name ....

Signature .... Date …………………………......

‘NAP SACC UK Research Study’
The Nutrition and Physical Activity Self Assessment for Child Care
Name of researcher: Dr Ruth Kipping

We are doing some research with nurseries to improve the health of young children. As part of this research we are inviting staff in nurseries which have taken part in the NAP SACC UK study to take part in an interview.

Would you be willing to take part?

INFORMATION FOR NURSERY STAFF CONSENT FOR INTERVIEW

What is the purpose of the research?
It is important for children to do some exercise and eat fruit and vegetables every day for healthy growth and development. Lots of children in England and Wales do not do enough exercise or eat enough fruit and vegetables. One in five children who start primary school are overweight or obese.

This study aims to test whether we can adapt and use a 6 month programme from the United States called “The Nutrition and Physical Activity Self Assessment for Child Care” (NAP SACC). We have made changes to use it in the UK and have tested whether we can work with nurseries to make them healthier places for children.

We would like to find out what nursery staff think about the NAP SACC UK study. As part of this research, you are being invited to participate in an interview with a researcher. The interview will take no more than 1 hour and will take place at a time which is convenient to you. It will take place over the phone or at your nursery – whichever you prefer. The information we gather will help us understand the thoughts and opinions of nursery staff who have been involved in the study. The information we collect will be used to help us decide how to use ‘NAP SACC UK’ in the future.

Who can take part?

Your participation in the interview is voluntary. You can choose not to take part, or you may withdraw at any time.

What are the risks?

There are no risks associated with taking part in the interview.

What are the benefits in taking part?

The information will help us decide what changes we need to make to the programme and the research study.

Will the information I give be confidential?

Everything you say during the interview will remain confidential. No information will be given to your nursery or other child care providers. No names or identifying information will be used in any results, publication or presentations. The interview session will be audio-taped. Direct quotes (things you have said) during the training may be used in reports, but any quotes will be anonymous. No identifying information will be included in quotes so that you cannot be identified (i.e. we will **not** include your name or the name of your employer).

The main circumstances under which the researchers would break confidentiality are where an individual is regarded to be at risk of serious harm. All participants will be informed that if they disclose information about neglect or abuse we will pass this information on to an appropriate source.

What do I do if I have questions?

If you have any further questions about the study, please contact the Principal Investigator, Dr Ruth Kipping, or one of the members of the NAP SACC UK study team on 0117 928 7308 or email napsacc-uk@bristol.ac.uk. They will be happy to assist with any queries.

What should I do next?

If you are happy to take part in an interview please complete the reply form on the next page, signing all relevant boxes and filling in the information requested. Return the form in the prepaid envelope enclosed. One of the research team will then contact you with the time and venue.

If you do not wish to take part in the interview you do not need to do anything, however you may receive a reminder letter in a few weeks. If you do not wish to take part please ignore this letter.

‘NAP SACC’ study

NURSERY STAFF INTERVIEW REPLY & CONSENT FORM

Name of lead researcher: Dr Ruth Kipping

Please complete all details and return in prepaid envelope:

Please put your initials in box for each question

I have read and understood the information sheet (version 2; dated 07/12/15)	☐
I do wish to take part in the interview and I am happy for the NAP SACC UK team to contact me about a time for the interview.	☐
The purpose of the interview has been explained to me, and I have been given the chance to discuss any questions or concerns with the researcher.	☐
I understand that the interview will be audio-taped but that my responses will remain confidential and I will not be able to be identified in any results.	☐
I agree to take part in the interview and understand that I may stop the interview at any time without giving a reason.	☐
I understand that the information collected will be used to support other research in the future, and may be shared anonymously with other researchers.	☐

Name of member of staff...

Job title ....

Name of nursery/child care provider ....

Address...

....

Telephone number...

Preferred time for interview: Morning ☐ Afternoon ☐ Evening ☐

Preferred place for interview: Face to face in Nursery ☐ By telephone ☐

Signature .... Date ………………………………

Please return form in prepaid envelope provided

NAP SACC UK, University of Bristol, Canynge Hall, 39 Whatley Rd, Bristol BS8 2PS

To be completed by researcher:

Researcher name ....

Signature .... Date …………………………....

‘NAP SACC UK Research Study’
The Nutrition and Physical Activity Self Assessment for Child Care

Name of researcher: Dr Ruth Kipping

We are doing some research with nurseries to improve the health of young children.

As part of this research we are inviting

NAP SACC UK Partners to take part in an interview.

Would you be willing to take part?

INFORMATION FOR CONSENT FOR NAP SACC UK PARTNER INTERVIEW

What is the purpose of the research?

It is important for children to do some exercise and eat fruit and vegetables every day for healthy growth and development. Lots of children in England and Wales do not do enough exercise or eat enough fruit and vegetables. One in five children who start primary school are overweight or obese.

This study aims to test whether we can adapt and use a 6 month programme from the United States called “The Nutrition and Physical Activity Self Assessment for Child Care” (NAP SACC). We have made changes to use it in the UK and have tested whether we can work with nurseries and parents to make nurseries and homes healthier places for children.

We would like to find out what NAP SACC UK Partners think about the NAP SACC UK study. As part of this research, you are being invited to participate in an interview with a researcher. The interview will take no more than 1 hour and will take place at a time which is convenient to you, either at your place or work or over the telephone. The information we gather will help us understand the thoughts and opinions of NAP SACC UK Partners who have been involved in the study. We will be asking what you think about the NAP SACC UK study. The information we collect will be used to help us decide how to use ‘NAP SACC UK’ in the future.

Who can take part?

If you have been involved in delivering the NAP SACC UK intervention to nurseries as a NAP SACC UK Partner you are able to take part in the interviews. You can choose not to take part, or you may withdraw at any time.

What are the risks?

There are no risks associated with taking part in the interview.

What are the benefits in taking part?

Information collected from the interviews will provide a better understanding of your experience of delivering the intervention to nurseries. The information will help us decide what changes we need to make to the programme and the research study in the future.

Will the information I give be confidential?

Everything you say during the interview will remain confidential. No information will be given to your employer or the child care providers. No names or identifying information will be used in any results, publication or presentations. The interview session will be audio-taped. Direct quotes (things you have said) during the training may be used in reports, but any quotes will be anonymous. No identifying information will be included in quotes so that you cannot be identified (i.e. we will **not** include your name or the name of your employer).

The main circumstances under which the researchers would break confidentiality are where an individual is regarded to be at risk of serious harm. All participants will be informed that if they disclose information about neglect or abuse we will pass this information on to an appropriate source.

What do I do if I have questions?

If you have any further questions about the study, please contact the Principal Investigator, Dr Ruth Kipping, or one of the members of the NAP SACC UK study team on 0117 9287308 or email napsacc-uk@bristol.ac.uk. They will be happy to assist with any queries.

What should I do next?

If you are happy to take part in an interview, please complete the reply form on the next page, signing all relevant boxes and filling in the information requested. Return the form in the prepaid envelope enclosed. One of the research team will then contact you to book a time which is convenient for you to do the interview.

If you do not wish to take part in the interviews you do not need to do anything, however you may receive a reminder letter in a few weeks. If you do not wish to take part please ignore this letter.

UK RESEARCH STUDY

University of BRISTOL

‘NAP SACC’ study

NAP SACC UK PARTNER INTERVIEW REPLY & CONSENT FORM

Name of lead researcher: Dr Ruth Kipping

Please complete all details and return in prepaid envelope:

Please put your initials in box for each question

I have read and understood the information sheet (version 2; dated 07/12/15)	☐
I do wish to take part in the parent interviews am happy for the NAP SACC to telephone me to arrange a convenient time for the interview	☐
The purpose of the interview has been explained to me, and I have been given the chance to discuss any questions or concerns with the researcher.	☐
I understand that the interview will be audio-taped but that my responses will remain confidential. Any use of direct quotes will be anonymised and I will not be able to be identified in any results.	☐
I agree to take part in the interview and understand that I may stop the interview at any time without giving a reason.	☐
I understand that the information collected will be used to support other research in the future, and may be shared anonymously with other researchers.	☐

Name of NAP SACC UK Partner……..……………………………………………………….....

Employing Organisation……………………………………………………………………….

Best telephone number to contact (including code) ……………………………….................

Preferred time for interview: Morning ☐ Afternoon ☐ Evening ☐

Preferred place for interview: Face to face at Nursery ☐ By telephone ☐

Signature .... Date ………………………………...

Please return form in prepaid envelope provided

NAP SACC UK, University of Bristol, Canynge Hall, 39 Whatley Rd, Bristol BS8 2PS

To be completed by researcher

Researcher name ....

Signature .... Date …………………………......

‘NAP SACC UK Research Study’
The Nutrition and Physical Activity Self Assessment for Child Care

Name of researcher: Dr Ruth Kipping

We are doing some research with nurseries to improve the health of young children. As part of this research we are inviting staff in nurseries which have taken part in the NAP SACC UK study to take part in an interview.

Would you be willing to take part?

INFORMATION FOR CONSENT FOR PARENT INTERVIEW

What is the purpose of the research?

It is important for children to do some exercise and eat fruit and vegetables every day for healthy growth and development. Lots of children in England and Wales do not do enough exercise or eat enough fruit and vegetables. One in five children who start primary school are overweight or obese.

This study aims to test whether we can adapt and use a 6 month programme from the United States called “The Nutrition and Physical Activity Self Assessment for Child Care” (NAP SACC). We have made changes to use it in the UK and have tested whether we can work with nurseries and parents to make nurseries and homes healthier places for children.

We would like to find out what parents think about the NAP SACC UK study. As part of this research, you are being invited to participate in an interview with a researcher. The interview will take no more than 1 hour and will take place at a time which is convenient to you. It will take place over the phone – we will phone you. The information we gather will help us understand the thoughts and opinions of parents whose children have been involved in the study. We will be asking what you think about the measurements, the NAP SACC UK study in the nursery and at home. The information we collect will be used to help us decide how to use ‘NAP SACC UK’ in the future.

Who can take part?

If you have a child who is aged 2-4 who spends time at a nursery which has taken part in the NAP SACC UK study you can take part in the interview. Your participation in the interview is voluntary. You can choose not to take part, or you may withdraw at any time. If you choose to participate you will be given a £10 shopping voucher ('high street' voucher which is suitable in many supermarkets and high street shops) to thank you for your time.

If you take part in a telephone interview, the researcher will arrange to call you at a time that is convenient to you. The phone call will be made from the researcher, so you will not be charged for the call on your telephone bill.

What are the risks?

There are no risks associated with taking part in the interview. As said above, you will be compensated for your time with a £10 shopping voucher.

What are the benefits in taking part?

Information collected from the interviews will provide a better understanding of knowledge and parents' attitudes about healthy eating, drinking, exercise and sedentary behaviour (inactive behaviour such as sitting and watching TV/playing computer games). The information will help us decide what changes we need to make to the programme and the research study in the future.

Will the information I give be confidential?

Everything you say during the interview will remain confidential. No information will be given to your nurseries and other child care provider. No names or identifying information will be used in any results, publication or presentations. The interview session will be audio-taped. Direct quotes (things you have said) during the training may be used in reports, but any quotes will be anonymous. No identifying information will be included in quotes so that you cannot be identified (i.e. we will **not** include your name or the name of your employer).

The main circumstances under which the researchers would break confidentiality are where an individual is regarded to be at risk of serious harm. All participants will be informed that if they disclose information about neglect or abuse we will pass this information on to an appropriate source.

What do I do if I have questions?

If you have any further questions about the study, please contact the Principal Investigator, Dr Ruth Kipping, or one of the members of the NAP SACC UK study team on 0117 9287308 or email napsacc-uk@bristol.ac.uk. They will be happy to assist with any queries.

What should I do next?

If you are happy to take part in an interview, please complete the reply form on the next page, ticking all relevant boxes and filling in the information requested. Return the form in the prepaid envelope enclosed. One of the research team will then contact you to book a time which is convenient for you to do the interview.

If you do not wish to take part in the interviews you do not need to do anything, however you may receive a reminder letter in a few weeks. If you do not wish to take part please ignore this letter.

UK RESEARCH STUDY

University of BRISTOL

‘NAP SACC’ study
PARENT INTERVIEW REPLY AND CONSENT FORM

Name of lead researcher: Dr Ruth Kipping

Please complete all details and return in prepaid envelope:

Please put your initials in box for each question

I have read and understood the information sheet (version 2; dated 07/12/15)	[ ]
I do wish to take part in the parent interviews am happy for the NAP SACC to telephone me to arrange a convenient time for the interview	[ ]
The purpose of the interview has been explained to me, and I have been given the chance to discuss any questions or concerns with the researcher.	[ ]
I understand that the interview will be audio-taped but that my responses will remain confidential. Any use of direct quotes will be anonymised and I will not be able to be identified in any results.	[ ]
I agree to take part in the interview and understand that I may stop the interview at any time without giving a reason.	[ ]
I understand that the information collected will be used to support other research in the future, and may be shared anonymously with other researchers.	

Name of parent.................……..………………………………………………………….......

Age of child at nursery/child care provider ……………………………………………......

Name of nursery/child care provider ………………………………………….....................

Best telephone number to contact (including code) …………………………………..................

Best time of day to contact you (please tick) am [ ] pm [ ] evening [ ]

Signature .... Date ………………………………...

Please return form in prepaid envelope provided

NAP SACC UK, University of Bristol, Canynge Hall, 39 Whatley Rd, Bristol BS8 2PS

To be completed by researcher

Researcher name ....

Signature .... Date …………………………….....
